# A call to protect patients, correctional staff and healthcare professionals in jails and prisons during the COVID-19 pandemic

**DOI:** 10.1186/s40352-020-00119-1

**Published:** 2020-07-02

**Authors:** Oluwadamilola T. Oladeru, Nguyen-Toan Tran, Tala Al-Rousan, Brie Williams, Nickolas Zaller

**Affiliations:** 1 Harvard Radiation Oncology Program, Harvard Medical School, Massachusetts General Hospital, Boston, MA USA; 2 The Australian Centre for Public and Population Health Research, Faculty of Health, University of Technology Sydney, Ultimo, NSW Australia; 3 Division of Infectious Diseases and Global Public Health, University of California San Diego, La Jolla, CA USA; 4 Divisions of Geriatrics, University of California San Francisco and Amend at UCSF, San Francisco, CA USA; 5 Department of Health Behavior and Health Education, Rural and Global Public Health Program, University of Arkansas for Medical Sciences, Fay W. Boozman College of Public Health, 4301 West Markham #820, Little Rock, AK 72205 USA

**Keywords:** Correctional officers, Healthcare workers, Jails, Prisons, COVID-19

## Abstract

This editorial describes why surge planning in the community must account for potential infection outbreaks in jails and prisons, and why incarcerated people and those in contact with them, including over 450,000 correctional officers and thousands of healthcare staff working in prisons, are at significant risk of COVID-19 exposure. We then explain how our nation’s jails and prisons will continue to serve as breeding grounds for devastating COVID-19 outcomes and offer specific guidance and a call to action for the immediate development of correctional healthcare strategies designed to protect the health and safety of patients and correctional and healthcare staff and the communities in which they are situated. Correctional officers and correctional healthcare professionals need the nation’s reassurance during this dire time that they will not be abandoned and further stigmatized for responding to the needs of incarcerated people. Our collective health depends on it.

**Letter to the Editor**

As nations continue to struggle to bring the COVID-19 pandemic under control, the number of cases and deaths across many parts of the globe continue to increase. The response efforts, and corresponding successes and failures, to protect the health of patients and healthcare professionals during this unprecedented pandemic has laid bare the inherent weaknesses in both healthcare infrastructure and public health preparedness in many nations. The United States’ uneven, piecemeal race to “flatten the curve” has highlighted its own weaknesses in its ability to respond to a pandemic. Nowhere are these weaknesses more apparent than in U.S. correctional facilities.

Incarcerated people and those in contact with them, including over 450,000 correctional officers and thousands of prison healthcare staff, are at significant risk of COVID-19 exposure. Key public health strategies to reduce viral transmission, such as social distancing and self-quarantine, are impossible in most facilities. Once COVID-19 enters prisons, it will spread like wildfire as conditions in prisons and jails, such as poor sanitation and overcrowded living conditions, make them breeding grounds for devastating outcomes. We have witnessed the beginnings of such devastation in prison and jail facilities across the U.S. One recent example is Marion County Correctional Institution which has reported that approximately three-quarters of its total incarcerated population (more than 2000 individuals) has tested positive for COVID-19 (Chappell & Pfleger, 2020).

The adverse health outcomes associated with unchecked spread of COVID-19 in prisons and jails will not remain inside its walls. Thousands of correctional staff exposed to COVID-19 will carry the infection back to communities such as rural jurisdictions where incarceration has risen by 26% since 2013 (Kang-Brown, Hinds, Schattner-Elmaleh, & Wallace-Lee, 2019). Many of these small communities have fewer hospital and intensive care beds per capita compared to urban settings (Schulte, Lucas, Rau, Szabo, & Hancock, 2020). A recent American Civil Liberties Union report suggests that more than 100,000 additional COVID-19 deaths could result if jail populations are not dramatically reduced (Knaack, 2020). As a result, we must take urgent action now to develop correctional healthcare strategies that protect the health and safety of patients, correctional staff and healthcare professionals. There are several immediate steps that should be taken to protect all who work or live in correctional facilities.

First, a dire shortage of personal protective equipment (PPE) for frontline healthcare and correctional staff poses a threat to their health and safety. In Ohio, nearly 250 correctional staff members have tested positive for COVID-19. Rikers Island Jail in New York City and Cook County Jail in Chicago have reported approximately 450 incarcerated people and 200 correctional staff, respectively, have been infected with COVID-19 (Lopez, 2020). Some of the cases reported in correctional facilities are in asymptomatic individuals and reflect the investment in broader testing efforts, especially where known outbreaks are occurring. However, this approach is not universal across the nation. More concerning is the public admission and fact that correctional facilities have a severe shortage of PPE (Barr, 2020) and infection rates will surge without their inclusion in statewide and county-level planning for PPE.

Second, correctional systems must prepare for enhanced respiratory support needs for more patients. Correctional facilities lack the healthcare infrastructure and equipment, such as ventilators, needed to manage advanced respiratory illness. Thoughtful planning for oxygen and basic life support supplies is critical. The capacity to offer bi-level positive airway pressure (BiPap) to a limited number of patients as a temporizing measure should be considered. Moreover, access to immediate and free on-site testing, treatment and care should be the standard in all facilities. Co-payment, which deters patients from seeking care, must be suspended.

Third, urgent attention to protect healthcare providers and correctional staff is needed to reduce COVID-19 transmission in both correctional facilities and communities. This is particularly important as many facilities do not allow basic cleaning or sanitation supplies that contain alcohol. Priorities include training all staff in basic knowledge about COVID-19 and its transmission, ways to minimize the risk of exposure, and clear instructions on the use of PPE. In addition, guidelines on access to hygiene products for both prison staff and residents should be liberalized, including increasing access to water, soap, hand sanitizer, and environmental sanitation and disinfection products.

Fourth, correctional officers are important resources in managing the anxiety and fears of those who are incarcerated. Policies intended to prevent officers from becoming too emotionally “close” to residents should be revised in this unique moment. Instead, correctional staff should be encouraged to provide emotional support to those residents with limited access to their family and friends on the outside.

Fifth, thoughtful development of public-health oriented decarceration strategies including accelerated release of those of older age and/or with chronic conditions should be prioritized (Simpson & Butler, 2020). However, decarceration of as many people as possible – whether or not they are in a particularly high-risk group, will help to enable social distancing among those who remain. As there are already thousands of releases every month from correctional facilities, emergency release planning teams must be developed and appropriately resourced for each facility to meet the increased need for the thoughtful skilled planning required to create appropriate housing and medical care plans.

The prevention and control of COVID-19 in our communities will only be as successful as our response to the most medically vulnerable populations, including people who are incarcerated. The widespread community transmission of COVID-19 has rendered the walls between correctional health and public health more permeable than ever. And even within the walls of our prisons and jails, everyone is connected. A recent tragic report from Louisiana describing a prison medical director, a head warden and an incarcerated individual all dying on the same day, highlights this fact (Skene, 2020). Correctional officers and healthcare providers need the nation’s reassurance during this dire time that they will not be abandoned and further stigmatized for responding to the needs of incarcerated people. Our collective health depends on it.

## Data Availability

Not Applicable.

## References

[CR1] Barr, L. (2020). *Federal prisons facing shortages of resources amid coronavirus outbreak*. ABC News https://www.vox.com/2020/4/22/21228146/coronavirus-pandemic-jails-prisons-epicenters. Accessed 23 Apr 2020.

[CR2] Chappell B, Pfleger P (2020). 73% of inmates at an Ohio prison test positive for coronavirus.

[CR3] Kang-Brown, J., Hinds, O., Schattner-Elmaleh, E., & Wallace-Lee, J. (2019). *People in jail in 2019*. Vera Institute of Justice https://www.vera.org/downloads/publications/people-in-jail-in-2019.pdf.

[CR4] Knaack, F. (2020). *Nearly 100,000 more people could die from COVID-19 in jails and surrounding communities if states fail to reduce jail populations. The time to act is now!* ACLU South Carolina https://www.aclusc.org/en/news/nearly-100000-more-people-could-die-covid-19-jails-and-surrounding-communities-if-states-fail. Accessed 23 Apr 2020.

[CR5] Lopez, G. (2020). *Why US jails and prisons became coronavirus epicenters*. Vox https://www.vox.com/2020/4/22/21228146/coronavirus-pandemic-jails-prisons-epicenters. Accessed 24 Apr 2020.

[CR6] Schulte, F., Lucas, E., Rau, J., Szabo, L., & Hancock, J. (2020). *Millions of older Americans live in counties with no ICU beds as pandemic intensifies*. Kaiser Health News https://khn.org/news/as-coronavirus-spreads-widely-millions-of-older-americans-live-in-counties-with-no-icu-beds/. Accessed 15 Apr 2020.

[CR7] Simpson PL, Butler TG (2020). COVID-19, prison crowding, and release policies. BMJ.

[CR8] Skene, L. (2020). *Coronavirus hits Louisiana prisons: medical director, head warden, first state inmate die*. The Advocate https://www.theadvocate.com/baton_rouge/news/coronavirus/article_697c5eb6-8354-11ea-a205-9726a420e972.html. Accessed 20 Apr 2020.

